# Observed hand cleanliness and other measures of handwashing behavior in rural Bangladesh

**DOI:** 10.1186/1471-2458-10-545

**Published:** 2010-09-09

**Authors:** Amal K Halder, Carole Tronchet, Shamima Akhter, Abbas Bhuiya, Richard Johnston, Stephen P Luby

**Affiliations:** 1 International Centre for Diarrhoeal Disease Research, Bangladesh, Dhaka, Bangladesh; 2 Water and Environmental Sanitation Section, UNICEF Bangladesh, Dhaka, Bangladesh; 3 Centers for Disease Control and Prevention, Atlanta, Georgia, USA

## Abstract

**Background:**

We analyzed data from the baseline assessment of a large intervention project to describe typical handwashing practices in rural Bangladesh, and compare measures of hand cleanliness with household characteristics.

**Methods:**

We randomly selected 100 villages from 36 districts in rural Bangladesh. Field workers identified 17 eligible households per village using systematic sampling. Field workers conducted 5-hour structured observations in 1000 households, and a cross-sectional assessment in 1692 households that included spot checks, an evaluation of hand cleanliness and a request that residents demonstrate their usual handwashing practices after defecation.

**Results:**

Although 47% of caregivers reported and 51% demonstrated washing both hands with soap after defecation, in structured observation, only 33% of caregivers and 14% of all persons observed washed both hands with soap after defecation. Less than 1% used soap and water for handwashing before eating and/or feeding a child. More commonly people washed their hands only with water, 23% after defecation and 5% before eating. Spot checks during the cross sectional survey classified 930 caregivers (55%) and 453 children (28%) as having clean appearing hands. In multivariate analysis economic status and water available at handwashing locations were significantly associated with hand cleanliness among both caregivers and children.

**Conclusions:**

A minority of rural Bangladeshi residents washed both hands with soap at key handwashing times, though rinsing hands with only water was more common. To realize the health benefits of handwashing, efforts to improve handwashing in these communities should target adding soap to current hand rinsing practices.

## Background

The WHO estimates that 3.8 million children aged under five die each year from diarrhoea and acute respiratory tract infections [1]. Intervention studies consistently demonstrate that communities that received intensive handwashing promotion have less childhood diarrhea and respiratory disease [2-5]. People in Bangladesh commonly believe that soap is not necessary for handwashing, that water alone is effective in purifying hands, especially when hands appear clean [6].

The Government of Bangladesh (GoB) and UNICEF with the support of the Department for International Development (DFID) of the British Government instituted the Sanitation, Hygiene Education and Water supply-Bangladesh project (SHEWA-B) in 2007. This project, which targets some 30 million underserved people, is among the largest intensive handwashing, hygiene/sanitation and water quality improvement programs ever attempted in a developing country. It aims to contribute towards achieving the Millennium Development Goals (MDGs) relating to water and sanitation.

Understanding usual handwashing practices in rural Bangladesh is an important baseline assessment for the program. One of the major challenges with assessing handwashing behavior is that no measure has proven to be both practical and valid [7,8]. Although structured observation of handwashing practices is widely considered the best available method, it is expensive, time-consuming, and risks being unrepresentative of usual handwashing practices [9]. Inspection of hands to assess their cleanliness has been suggested as an indicator of hand hygiene [10,11] but there are limited data that assesses the relationship of hand cleanliness to other handwashing indicators [12,13].

This evaluation assessed the pre-intervention baseline of handwashing practices across rural Bangladesh. The objective of this analysis was to describe typical handwashing practices in rural Bangladesh and assess the association of measures of hand cleanliness with hygiene associated household characteristics.

## Methods

### Study population

In the initial two years from 2007, the SHEWA-B project targeted interventions for 19.6 million underserved populations from 68 sub-districts (*upazilas*) in 19 districts. The method for selecting the evaluation population has been described previously [14]. Briefly, we listed all unions in the 68 sub-districts and selected 50 unions (clusters) randomly using the population proportion to size of unions. The union is the lowest administrative rural unit within the Government of Bangladesh. Each union typically includes 25-50 villages and each village typically includes 50 - 200 or more households. UNICEF and the Department of Public Health Engineering (DPHE) of the Government of Bangladesh selected non-intervention matched control sub-districts that had no major hygiene promotion intervention, but had similar infrastructure, agricultural productivity, household construction and hydrogeology for each randomly selected intervention sub-district. Fifty (50) unions within the control from 50 sub-districts were selected using the same population proportional to size method used to select intervention unions. In this way, the study selected 100 unions from 36 districts (out of 64) in Bangladesh.

Once the study randomly selected a union, the ICDDR,B field team secured a list of all villages from the government authority, assigned a number to each village, and used a random number table to select a village. The team identified the starting point within the village by assessing the center point with the help of village residents. They enrolled the first eligible household by identifying the closest household to the village center who had at least one child under the age of 5 years and consented to participate in the evaluation. To enroll the next household, fieldworkers skipped the next two closest households, and then looked for the next closest eligible household. The field team repeated the process of enrolling the next closest household for the cross sectional survey until they enrolled 17 households. The field team conducted structured observation in the initial 10 households. Thus the field team targeted 1000 households for structured observation and 1700 households for the cross sectional survey. Since SHEWA-B interventions had not been initiated at the time of the baseline assessment and we were interested in measures of handwashing behavior and associated hand hygiene indicators throughout rural Bangladesh, we included both the intervention and control households in this analysis.

### Instruments and data collection

Trained field data collectors conducted 5-hour structured observations between 9:00 AM and 2:00 PM during July-August 2007 in the selected households. Data collectors observed and recorded the handwashing behaviors of all available household members at pre-specified key times-- before eating, before feeding a child, after defecation and after cleaning a child who had defecated.

Two months later during October-December 2007, the field data collectors conducted a cross sectional survey that included individual interviews and spot checks. Data collectors used a structured questionnaire to interview the main caregiver of the youngest child at the household. After concluding the interview data collectors conducted spot checks, that is structured inspection of household latrines, handwashing stations to assess the availability of water and soap, and hand cleanliness for both mother/caregiver and children.

For measuring handwashing behaviors, besides structured observations, during the cross sectional survey, data collectors asked different key occasion/event specific questions for reported behavior of handwashing measurement; for instance, they asked to the respondents '*during the latest occasion after a defecation event in the preceding 24 hours, did you wash your hands with water and soap?*' The data collectors also asked the mothers/caregivers and one 3 - 5 year old child in the household to demonstrate how they usually washed their hands after defecation.

During the cross sectional survey the study workers assessed hand cleanliness for mothers/caregivers of the youngest children of all sampled households and for all under-5 children if available. Data collectors assessed the palm and finger pads of both hands and coded them as unclean if any visible dirt was seen and clean if there was no visible dirt. If at least one child in the household had at least one hand that was dirty, then for analysis we considered the household as a household with dirty child hands. To ensure uniform understanding among all data collectors, we provided in-house training for data collectors and field supervisors, conducted practical role play exercise and thereafter conducted pre-testing in the field with the data collection instruments for two days.

### Quality control of data collection

To maintain the quality of data collection the study recruited full time experienced field supervisors. Both field supervisors and data collectors were trained for one week on the study instruments and participated in two rounds of field testing. During data collection field supervisor randomly selected at least 5% of forms per day, re-visited the households, and checked the accuracy of the responses. At the end of each day, field supervisors rechecked all questionnaires and sent the data collectors back to the house to correct any inconsistencies within the form. The research coordinator, one co-investigator, two medical officers and an international research fellow made regular visits to each field team where they directly observed the interviewing techniques, checked completed questionnaires, and in order to improve the quality of data collection, provided immediate feedback on strengths and weaknesses of data collections to the data collectors.

### Data analysis

The primary outcome variables for structured observation data were whether a person under observation in the household had washed both hands using water alone or with soap at four key times: before eating, before feeding a child, after defecation and after cleaning a child who defecated. We calculated the proportion of different handwashing events using water and/or soap for structured observations at different key times. For the cross sectional survey we calculated proportions of caregivers and proportion of children who reported washing both hands using water alone and/or soap.

The primary outcome variables for the spot check were hand cleanliness for mother/caregiver and children aged 3-5 years.

To assess the relationship of observed hand cleanliness to household characteristics we calculated odds ratios. We evaluated the association between specific characteristics and hand cleanliness of mothers and children separately. We chose specific characteristics that previous studies suggested were associated with handwashing behavior including caregivers' handwashing behavior (observed), availability of separate soap for handwashing, availability of spare soap at household, use of improved latrines, availability of handwashing locations, availability of water and soap at handwashing locations, and respondents' economic status [7,11,14,15]. Besides respondents' self defined economic status, we measured household wealth by applying the principal component analysis (PCA) technique as scores of the first principal component characterizes economic status of sampled households [16]. To account for the clustering of observations in villages we used generalized estimated equations to calculate odds ratios and 95% confidence intervals [17].

To construct multivariate models we included as covariates those variables significantly associated (p < 0.05) with the outcome. We used an exchangeable correlation structure for all generalized estimated equations analyses to account for the clustering measures within different villages. We used STATA for Windows, Version SE/10 (STATA Corp, USA) for the generalized estimated equations modeling.

### Ethics

The data collectors sought informed consent before enrolling a household in the evaluation. In seeking consent, the data collectors described the objective of the evaluation, and noted the benefit of the intervention program and its evaluation for the rural Bangladeshi communities. Data collectors clarified that there was no special benefit to participating but also no health risks. They noted that confidentiality of data would be maintained, that participation for the study was voluntary, and refusing to participate would not affect the family getting any intervention services. Because this was an evaluation of a public health program rather than a primary research project, the program evaluation was not reviewed by a human subjects research panel i.e. we did not seek a waiver from a human subjects research panel.

## Results

The field team completed structured observations in 1000 households (100%) and cross sectional surveys in 1692 (99%) of the 1700 targeted households. Among the households in the cross sectional survey, 671 (40%) had a child aged 3 to 5 years old. The field team assessed a handwashing demonstration for 946 (93%) of 1021 targeted mothers/caregivers and 203 (30%) of 671 targeted 3-5 year old children. The other children were unwilling or unable to demonstrate handwashing. The field staff evaluated hand cleanliness of mothers/caregivers for 100% of evaluated households (1692) and for 96% of targeted children (1628).

The majority of households (94%) were headed by a male. Approximately one third of mothers (31%) and fathers (37%) lacked formal education. Almost half (45%) of the households had an electricity connection and 29% owned a mobile phone (table 1).

**Table 1:** Socio-demographic characteristics of respondents and households, rural Bangladesh, 2007 (N = 1692)

Characteristics	percent/mean/median (n)
Household head (%)	
Male	94 (1592)

Education of mother of the youngest child (%)	
No education	31 (525)
Up to primary	33 (565)
Above primary	36 (602)

Education of father of the youngest child (%)	
No education	37 (617)
Up to primary	31 (519)
Above primary	32 (556)

Occupation of father of the youngest child (%)	
Farmer/cultivator/homemaker	24 (409)
Agri & non-agri labor/boatman/shoe or umbrella mechanic	21 (357)
Traders/business occupation	18 (312)
Skilled worker/profession	10 (163)
Service	10 (161)
Rickshaw/Van puller	9 (143)
Staying abroad	6 (110)
Household head untraced, disabled, domestic maid, retired, unemployed	2 (37)

Mean number of household members	5.5 (1692)

Mean number of under-5 children per household	1.3 (1692)

Social status of households (Respondents' self assessment) (%)	
Rich	1 (17)
Upper middle	3 (48)
Middle	46 (776)
Poor	41 (693)
Hardcore poor	9 (158)

Ownership of living house (%)	
Self-owned	94 (1584)
Others (Rental, Govt. land, owned by a landlord, relative house)	6 (108)

Median amount of homestead land (sq. meter)	280 (1692)

Median amount of land other than homestead (sq. meter)	260 (1692)

Median number of sleeping room	2 (1692)

Households own (%)	
Electricity	45 (762)
Almirah/wardrobe	27 (459)
Television (B/W)	19 (322)
Television (color)	9 (147)
Refrigerator	2 (39)
Motor cycle	2 (38)
Mobile phone	29 (498)

During structured observation based on all key times, most study subjects either did not wash any hand (42%) or washed one hand only (46%). Among those who did wash both hands, most washed with only water before eating, before feeding a child, after defecation and after cleaning a child who defecated (Table 2). Use of soap while washing both hands was less common compared to washing hands with only water. For food related events such as before eating and before feeding a child, people used soap for washing both hands less than 1% of the time, whereas soap was used for handwashing more commonly after defecation (14%) and after cleaning a child who defecated (21%). Adult caregivers (mainly female) were more likely to wash both their hands with soap than children (Table 2).

**Table 2:** Structured observation: handwashing measures at different critical times, Rural Bangladesh, 2007

Indicators	Washed both hands before eating % (n)		Washed both hands before feeding a child % (n)		Washed both hands after defecation % (n)		Washed both hands after cleaning a child who defecated % (n)	
	**with only water**	**with water and soap**	**with only water**	**With water and soap**	**With only water**	**With water and soap**	**With only water**	**With water and soap**

Adult caregiver	7 (86)	0.5 (6)	4 (68)	1 (16)	18 (13)	34 (24)	23 (86)	21 (79)

Children: 3-5 years	2 (26)	0	0	0	17 (9)	6 (3)	100 (2)	0

Children: 5-12 years	4 (56)	0.4 (6)	4 (1)	0	10 (9)	7 (6)	0	0

Male non-caregiver (12 years and above)	5 (62)	0.5 (6)	3 (1)	0	25 (23)	10 (9)	22 (2)	0

Female non-caregiver (12 years and above)	6 (42)	1.1 (8)	5 (3)	0	48 (10)	18 (8)	19 (5)	27 (7)

All Persons (3+ years)	5 (272)	0.4 (26)	4 (73)	0.9 (16)	18 (64)	14 (50)	23 (95)	21 (86)

The various methods of assessing handwashing measured markedly different frequencies of handwashing practices (Table 3). When field workers asked mothers/caregivers and children to demonstrate their normal handwashing after defecation, 51% of mothers/caregivers and 37% of children used water and soap together and rubbed both hands. The proportion of caregivers who washed their both hands with soap when asked to demonstrate how they wash their hands after defecation (51%) was similar to their reported behavior (47%), but much higher than was observed during structured observation (33%) (Table 3).

**Table 3:** Handwashing measures at different critical times by different methods, Rural Bangladesh, 2007

Indicators	Before eating % (n)	Before feeding a child % (n)	After defecation % (n)	After cleaning a child who defecated % (n)
Washed both hands with only water				

Structured observation: Female caregivers (events)	7 (84)	4 (68)	19 (13)	23 (86)

Respondents' report: During last occasion prior to survey date (N = 1692)	30 (512)	16 (273)	10 (166)	13 (216)

Handwashing Demo: Caregivers (N = 946)	-	-	10 (97)	-

Handwashing Demo: Children (3-5 years age) (N = 203)	-	-	16 (32)	-

Washed both hands with water and soap				

Structured observation: Female caregivers (events)	0.5 (6)	1 (16)	33 (23)	22 (79)

Respondents' report: During last occasion prior to survey date (N = 1692)	12 (211)	9 (160)	47 (801)	43 (735)

Handwashing Demo: Caregivers (N = 946)	-	-	51 (482)	-

Handwashing Demo: Children (3-5 years age) (N = 203)	-	-	37 (75)	-

Spot checks during the cross sectional survey classified 930 caregivers (55%) and 453 children (28%) as having clean appearing hands. In bivariate analysis, 5 of 8 household characteristics--spare soap available at household, use of an improved latrine, water available at handwashing locations, soap available at handwashing location and economic status, were associated with mothers' hand cleanliness. These same five household characteristics plus having separate soap available at the household for handwashing was also associated with child hand cleanliness in bivariate analysis (Table 4).

**Table 4:** Bivariate analysis: Characteristics associated with hand cleanliness (clean palms and finger pads) for mothers/caregivers and young children (N = 1692 households for cross sectional survey, N = 997 households for structured observation)

Characteristics	Caregivers' with this characteristic and clean palms and finger pads % (n)	Caregivers' without this characteristic and clean palms and finger pads % (n)	Adjusted OR* (95% CI), p value	Children with this characteristic and clean palms and finger pads % (n)	Children without this characteristic and clean palms and finger pads % (n)	Adjusted OR* (95% CI), p-value
Female caregivers ever washed hands with soap (observed)	56 (84)	53 (446)	1.07 (0.72, 1.56), p = 0.698	29 (41)	25 (201)	1.21 (0.81, 1.79), p = 0.356

Separate soap available at household for handwashing (spot checked)	53 (215)	55 (715)	0.91 (0.73, 1.14), p = 0.432	33 (128)	26 (325)	1.41 (1.10, 1.81), p = 0.006

Spare soap available at household (spot checked)	60 (324)	53 (606)	1.34 (1.08, 1.64), p = 0.006	33 (168)	25 (285)	1.41 (1.12, 1.78), p = 0.003

Used improved latrine (spot checked the infrastructure)	61 (241)	53 (689)	1.40 (1.11, 1.77), p = 0.004	35 (130)	26 (323)	1.55 (1.21, 1.99), p = 0.001

Handwashing location after toilet use: within 10 feet (spot checked)	54 (627)	58 (303)	0.83 (0.70, 1.06), p = 0.150	29 (323)	25 (130)	1.22 (0.96, 1.55), p = 0.104

Water available at handwashing locations after toilet use (spot checked)	58 (713)	46 (217)	1.60 (1.29, 1.98), p = 0.000	31 (360)	20 (93)	1.72 (1.32, 2.24), p = 0.000

Soap available at handwashing locations after toilet use (spot checked)	58 (493)	52 (437)	1.29 (1.06, 1.57), p = 0.010	32 (261)	24 (192)	1.54 (1.24, 1.93), p = 0.000

Self defined economic status: middle class and above	61 (511)	49 (419)	1.59 (1.31, 1.93), p = 0.000	33 (266)	23 (187)	1.70 (1.36, 2.12), p = 0.000

Economic status based on Principal Component Analysis (PCA)						

2^nd ^quintile (compared to poorest)	49 (165)	47 (159)	1.07 (0.79, 1.44), p = 0.662	21 (68)	19 (63)	1.11 (0.76, 1.63), p = 0.595
3^rd ^quntile (compared to poorest)	57 (194)	47 (159)	1.50 (1.11, 2.04), p = 0.009	27 (90)	19 (63)	1.64 (1.13, 2.37), p = 0.009
4^th ^quintile (compared to poorest)	59 (199)	47 (159)	1.64 (1.20, 2.24), p = 0.002	37 (119)	19 (63)	2.47 (1.71, 3.55), p = 0.000
5^th ^quintile (compared to poorest)	63 (213)	47 (159)	1.92 (1.41, 2.61) p = 0.000	35 (113)	19 (63)	2.35 (1.64, 3.36), p = .000

In multivariate analysis, two of these variables -- household wealth as assessed by principal component analysis and water availability at handwashing locations - remained significantly associated with the hand cleanliness for both mother and child (table 5). The odds ratios in the multivariate analysis were little changed from the bivariate analysis.

**Table 5:** Multivariate analysis* of characteristics associated with hand cleanliness for mothers/caregivers and children, Rural Bangladesh, 2007

Characteristic	Mothers/caregivers		Children	
	Adjusted Odds ratio* (95% Confidence Limit)	P-value	Adjusted Odds ratio* (95% Confidence Limit)	P-value
Separate soap available at household for handwashing (spot checked) compared to not available^†^			1.16 (0.89, 1.50)	0.262

Spare soap available at household (spot checked) compared not available	1.13 (0.90, 1.41)	0.297	1.08 (0.84, 1.38)	0.547

Used improved latrine (spot checked the infrastructure) compared to unimproved latrine	1.14 (0.89, 1.45)	0.307	1.17 (0.89, 1.53)	0.261

Water available at handwashing locations after toilet use (spot checked) compared to not available	1.38 (1.10, 1.73)	0.006	1.34 (1.01, 1.77)	0.042

Soap available at handwashing locations after toilet use (spot checked) compared to not available	1.08 (0.88, 1.32)	0.469	1.21 (0.95, 1.53)	0.121

PCA constructed economic status: Economic score is a ordinal variable (quintiles as 1 (poorest), 2, 3, 4, 5 (Richest))	1.12 (1.04, 1.21)	0.004	1.20 (1.09, 1.31)	0.000

*Odds ratios were calculated using a generalized estimated equations model that accounted for neighborhood clustering using an exchange correlation structure

^†^We didn't include this variable during multivariate analysis with mothers hand cleanliness there was no significant association in the bivariate analysis

## Discussion

Residents of these rural Bangladeshi communities commonly washed their hands with only water, but they infrequently washed both hands with soap. Similar findings have been noted in other settings [18,19]. Since interventions that promote handwashing with soap have resulted in reduced diarrheal and respiratory disease [2,3], behavior change programs should build upon people's normal hand rinsing behavior to add the use of soap [20].

Although handwashing with soap after fecal contacted events is dangerously low, it is much higher than handwashing before feeding a child or eating. Bangladeshi residents conceptualize the need for handwashing quite differently after fecal contact than before feeding a child or eating [6]. Behavioral change intervention for these two different contexts may need to be different. The relative impact on population health of focusing behavior change interventions solely on handwashing after fecal contact rather than on both fecal and food related contact is unknown.

Although handwashing with soap after fecal contact was more common among mothers/caregivers than other categories of household members; but similar to findings in other studies [21,22], this study also found that most mothers/caregivers failed to wash their hands with soap. Also like other studies [23,24], reported handwashing with soap was much higher than the observed behavior. Compared to observed practice, a higher proportion of people demonstrated the skill of both handwashing with soap after defecation. This proportion is much higher than in a similar assessment of 120 households in Panmana (Rural Kerala- India) in which only 10% of controls in a sanitation study demonstrated handwashing using soap and water and rubbing both hands together on request [25]. Nevertheless, the higher level of knowledge and skill among the study population in rural Bangladesh did not lead to optimal handwashing practices.

Hand cleanliness as assessed by field workers is a potential low cost method to evaluate hand hygiene practices. In this evaluation observed hand cleanliness of the palms and finger pads was independently associated with wealth and the presence of water at the most convenient place to wash hands in household. Indicators of handwashing are commonly strongly associated with measures of socioeconomic status [15,26,27]. In an earlier analysis from this same SHEWA-B baseline evaluation, the presence of water at their most convenient place to wash hands was independently associated with a doubling of the likelihood of handwashing with soap after fecal contact as observed in structured observation [14]. Although hand cleanliness was associated with water at a handwashing station, it was not associated with observed handwashing behavior as measured by structured observation. The independent association between hand cleanliness and the presence of water at their most convenient place to wash hands suggests that hand cleanliness may be an independent marker of handwashing behavior. In a study in Tanzania resulted visibly dirty hands were associated with microbiological indicators of fecal contamination [12]. Further research on this potential indicator would be useful.

The observed behavior of child mothers/caregivers within this study may not represent their actual behavior because the presence of an observer can change the handwashing behavior of subjects [9,28]. We attempted to reduce the impact of the observer by conducting observations for extended 5-hour periods rather than shorter time intervals that have been associated with increased reactivity [26,29]. A second limitation of this study is that we did not assess inter-rater reliability of data collectors' assessments of hand cleanliness. Although the method of training we used was feasible for a program evaluation, formal evaluation of inter-rater reliability would be an important component of future research using this method. A third limitation of this analysis is that the populations were specifically targeted as under served rural communities, and hence may not represent the broader rural population in Bangladesh. However, the study represents the high need population of rural Bangladesh.

## Conclusions

The proportion of Bangladeshi residents in these rural communities who washed their hands with soap at key times was consistently low. There is a deeply-rooted belief among Bangladeshi people that water is a potent purifying agent. Accordingly, hands which have been rinsed with water alone and which show no visible contamination are considered to be clean [6]. Moreover, people who wash hands with soap after defecation mainly do so to remove the unpleasant fecal odour [30]. Thus, washing hands with only water before eating seems sufficient as noted by the <1 percent of people observed washing hands with soap before eating. In Ghana a multi-channel communication handwashing interventions successfully conveyed the message that hands were not truly clean unless washed with soap [20]. Efforts to improve handwashing with soap in Bangladesh will need to directly address this belief on the importance of soap in order to improve handwashing behavior and unlock the potential of this public health intervention.

## Competing interests

The authors declare that they have no competing interests.

## Authors' contributions

Conception and design of the study: AKH, CT, AB, RJ, SA, SPL; Analysis and interpretation of data: AKH, SPL; Writing paper: AKH, SPL; Contribution to reagents/materials: AB, CT, RJ, SA. All authors read and approved the final manuscript.

## Pre-publication history

The pre-publication history for this paper can be accessed here:

http://www.biomedcentral.com/1471-2458/10/545/prepub
